# BMP-7 Does Not Protect against Bleomycin-Induced Lung or Skin Fibrosis

**DOI:** 10.1371/journal.pone.0004039

**Published:** 2008-12-29

**Authors:** Lynne A. Murray, Tillie L. Hackett, Stephanie M. Warner, Furquan Shaheen, Rochelle L. Argentieri, Paul Dudas, Francis X. Farrell, Darryl A. Knight

**Affiliations:** 1 Immunobiology Department, Tissue Remodeling and Metabolism Department, Centocor, Radnor, Pennsylvania, United States of America; 2 James Hogg iCapture Centre for Cardiovascular and Pulmonary Research, St Paul's Hospital, Vancouver, Canada; 3 Department of Anesthesiology, Pharmacology and Therapeutics, University of British Columbia, Vancouver, Canada; University of Giessen Lung Center, Germany

## Abstract

Bone morphogenic protein (BMP)-7 is a member of the BMP family which are structurally and functionally related, and part of the TGFβ super family of growth factors. BMP-7 has been reported to inhibit renal fibrosis and TGFβ1-induced epithelial-mesenchymal transition (EMT), in part through negative interactions with TGFβ1 induced Smad 2/3 activation. We utilized *in vivo* bleomycin-induced fibrosis models in the skin and lung to determine the potential therapeutic effect of BMP-7. We then determined the effect of BMP-7 on TGFβ1-induced EMT in lung epithelial cells and collagen production by human lung fibroblasts. We show that BMP-7 did not affect bleomycin-induced fibrosis in either the lung or skin *in vivo*; had no effect on expression of pro-fibrotic genes by human lung fibroblasts, either at rest or following exposure to TGFβ1; and did not modulate TGFβ1 -induced EMT in human lung epithelial cells. Taken together our data indicates that BMP-7 has no anti-fibrotic effect in lung or skin fibrosis either *in vivo* or *in vitro*. This suggests that the therapeutic options for BMP-7 may be confined to the renal compartment.

## Introduction

Heightened activation, altered phenotype and augmented synthetic activity of collagen producing cells are hallmarks of fibrosis. In the lung, fibrotic diseases such as idiopathic pulmonary fibrosis (IPF), result in reduced lung compliance and loss of alveolar architecture, which ultimately leads to organ failure and death of the afflicted individual [1], [2]. Similarly, in the skin, a significant population of people affected by diseases such as scleroderma also have substantial fibrosis of the lungs and other internal organs. The progressive decline in organ function in these diseases is pronounced and unrelenting, due, in part, to the lack of effective treatments being available.

TGFβ1, is the prototypical pro-fibrotic cytokine and is over-expressed in fibrosis in multiple human settings and animal models [3]–[5]. This growth factor induces excess extracellular matrix (ECM) generation, enhanced fibroblast survival and the differentiation of fibroblasts to αSMA-positive myofibroblasts, which are relatively absent from normal lungs [1]–[3]. Additionally, one of the more recent activities ascribed to TGFβ1 is epithelial to mesenchymal transition (EMT) [4]–[6]. EMT is a dynamic process by which epithelial cells undergo phenotypic transition to motile mesenchymal cells such as fibroblasts and myofibroblasts and is accompanied by downregulation of epithelial proteins such as E-cadherin with a concomitant increase in mesenchymal cell markers such as vimentin and EDA-fibronectin [7], [8]. EMT has been observed in several experimental models of fibrosis where it has been shown to account for up to 20% of the interstitial fibroblasts [9]–[12]. Several recent studies have shown that EMT occurs in lung epithelial cells both *in vitro* and *in vivo*, supporting the concept of EMT contributing to the fibrosis observed in IPF [5], [13], [14]. However, intracellular processes that modify fibrotic pathways and EMT in particular, have not been evaluated.

Bone morphogenic protein (BMP)-7 is a member of the BMP family which are structurally and functionally related, and part of the TGFβ superfamily of growth factors [15]. There are three type I and three type II receptors so far identified and any two of each forms a heterotetramer receptor that BMP-7 can signal through [16], [17]. Binding of BMP-7 to its receptor complex results in recruitment and phosphorylation of Smad's 1, 5 and 8 [15], [18], which then associate with Smad 4, translocate to the nucleus and confer gene expression [19], [20]. BMP-7 has also been shown to modulate TGFβ1 signaling, by either inhibiting Smad 2/3 phosphorylation or by direct competition for Smad 4, or both [19], [20]. Accordingly, BMP-7 has also been reported to inhibit many processes involving TGFβ1 including renal fibrosis [21], cardiac fibrosis and allograft rejection *in vivo* [22] as well as EMT and at high concentrations, endothelial cell-mesenchymal transition [22]. However, in other cells, such as hepatocytes or breast epithelial cells, BMP-7 appears to have either no effect [8] or the opposite effect [23], suggesting some organ specificity. In the present study we sought to evaluate the role of BMP-7 in lung and skin fibrosis as well its capacity to regulate the pro-fibrotic effects of TGFβ1 *in vitro*.

## Results

### BMP-7 does not inhibit intratracheal bleomycin-induced pulmonary fibrosis

**Figure 1:**
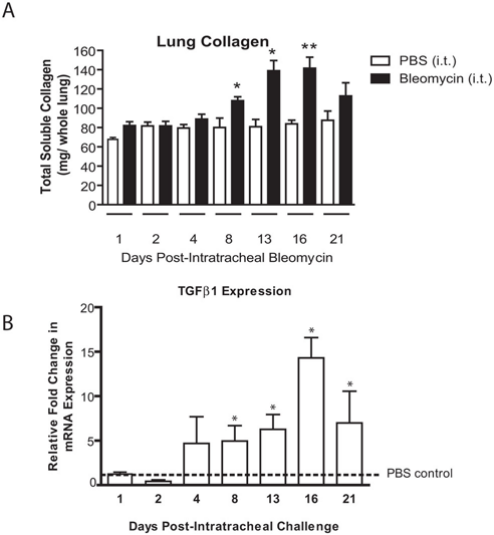
Timecourse of bleomycin-induced lung fibrosis. (A) Timecourse of induction of collagen protein generation in the lungs of mice challenged with intratracheal bleomycin (filled bars) or intratracheal PBS (open bars) as measured by the Sircol Assay (mean±S.E.M. of n = 4–8 mice/group). (B) Real-time RT-PCR analysis of TGFβ_1_ (open bars) gene expression in the lungs of mice challenged with intratracheal bleomycin compared to PBS control (dotted line) at the same corresponding time points (mean value of n = 3 mice/group). For differences between bleomycin challenged and corresponding time point PBS control groups * indicates p<0.05, **p<0.01 statistical significance difference between bleomycin and corresponding time point PBS control.

In initial experiments we correlated collagen deposition in the lung following bleomycin administration, with the expression of TGFβ1. Mice were treated with either bleomycin or saline as a control on day 0, with cohorts being sacrificed at days 1, 2, 4, 8, 13, 16 and 21 after intratracheal challenge. There was a significant increase in total collagen deposition observed as early as 8 days post-bleomycin which continued to increase until day 16, after which levels began to decrease (Fig. 1A). Gene transcription of TGFβ1 followed a similar pattern of induction with significant increases being noted at day 4, continuing to a maximum after 16 days and decreasing at day 21 (Fig. 1B).

Due to the reported effects of BMP-7 inhibiting TGFβ1 pathway, we systemically administered BMP-7 (500 µg/kg) to mice daily for 2 weeks. This dose level is higher than previously reported to be efficacious [21]. Therefore the amount used in the *in vivo* studies were supramaximal than anticipated efficacious dose levels.

**Figure 2:**
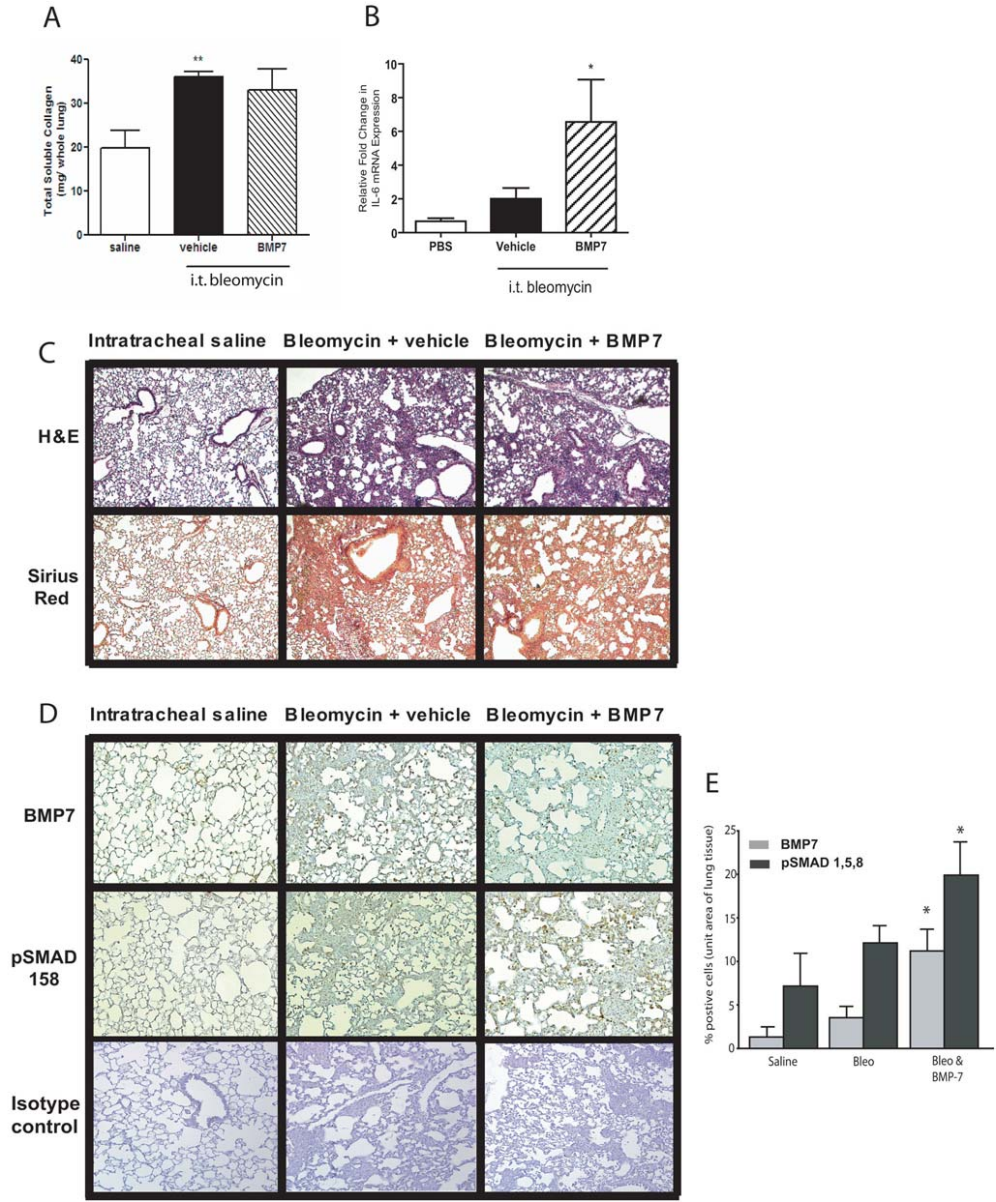
BMP-7 does not inhibit intratracheal bleomycin-induced pulmonary fibrosis. (A) Total soluble collagen levels in lung homogenates from mice challenged with bleomycin or PBS (open bars) 14 days post-intratracheal administration, and treated with vehicle (filled bars) or BMP-7 (hatched bars) daily (mean±S.E.M. of n = 5 mice/group). (B) Real-time RT-PCR analysis of IL-6 gene expression in the lungs of mice challenged with intratracheal PBS (open bars) or bleomycin and treated daily with BMP-7 (hatched bars) or vehicle (filled bars) for 14 days (mean±S.E.M of n = 3 mice/group). Representative photomicrographs of sections of lungs from mice 14 days after intratracheal delivery of saline (left) or 14 days after intratracheal bleomycin and treatment with daily injections of BMP-7 (right) or vehicle control (middle). Sections are stained with (C) haematoxylin and eosin (top panels) or PicroSirius red (bottom panels); or immunostained for (D) BMP7 (top panels) or phosphor- Smad 1,5,8 (middle panels) expression or isotype control (bottom panels) Fields viewed at ×200 magnification. (E) Quantification of immunostaining for the number of positive cells for BMP7 (grey bars) or phosphor- Smad 1,5,8 (black bars) in lung sections using image pro©. Bars represent mean number of positive cells±S.E.M. for three sections. For differences between bleomycin and corresponding PBS control, * indicates p<0.05, **p<0.01 statistical significance.

As peak collagen synthesis occurred between day 13 and 16, mice were sacrificed on day 14 and lungs analyzed for collagen production and expression of pro-fibrotic genes. As seen in Figure 2A, we found that BMP-7 had no effect on bleomycin-induced lung collagen deposition. Administration of rhBMP-7 also induced the expression of IL-6 mRNA in lung tissue (Fig. 2B). To confirm that BMP-7 did not alter the deposition of collagen or gross pathology induced by bleomycin, we also histologically analyzed lungs from all cohorts of animals. As expected, bleomycin induced disorganization of normal alveolar architecture, pronounced collagen accumulation and interstitial infiltrates. Moreover, comparable histopathology was observed in the BMP-7-treated mice (Fig. 2C). We confirmed that BMP-7 reached the lungs we stained lung sections with an anti-BMP-7 antibody (Fig. 2D). Moreover BMP-7 was biologically active in the lungs of dosed mice as we observed increased phosphorylation of Smad 1,5,8 (Fig. 2D and E). Taken together, this indicated that BMP-7 had no protective or inhibitory effect on the fibrotic response induced by intratracheal bleomycin.

### Systemic BMP-7 does not inhibit subcutaneous bleomycin-induced skin or lung collagen

**Figure 3:**
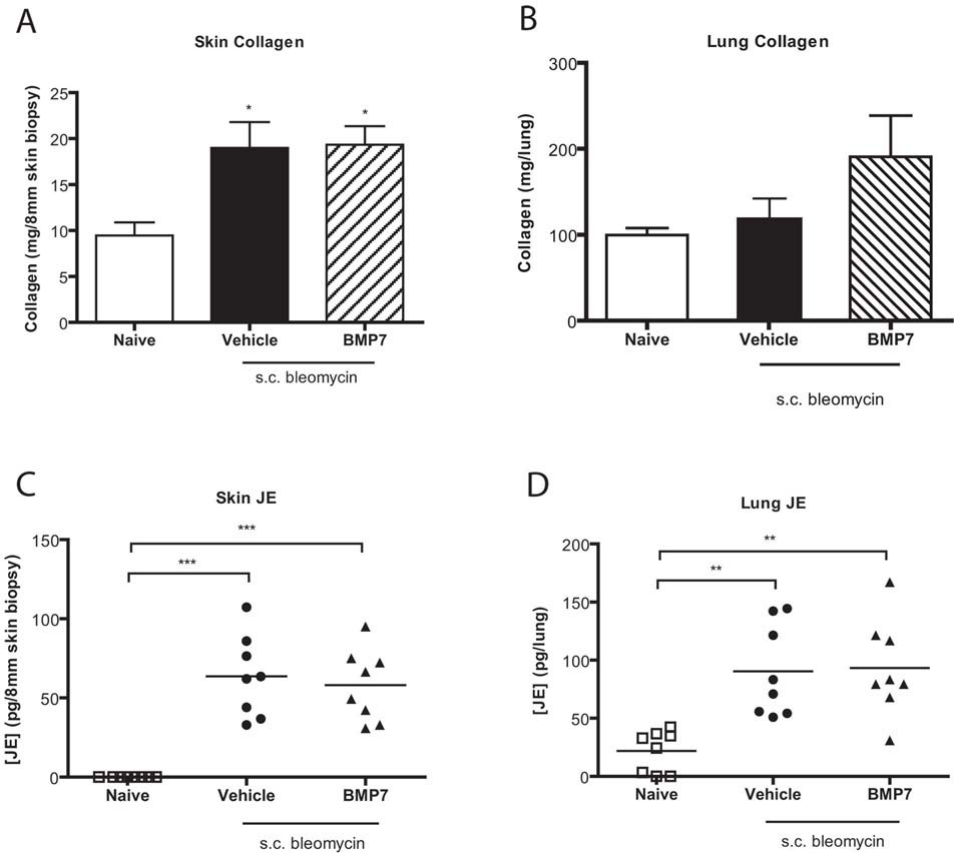
BMP-7 does not inhibit subcutaneous bleomycin-induced skin and lung fibrosis. Total collagen in the (A) skin or (B) lungs of mice challenged daily with subcutaneous bleomycin and treated daily with either BMP-7 (hatched bars) or vehicle control (filled bars) (mean±S.E.M. of n = 8 mice/group). JE/CCL2 protein levels were measured by luminex in (C) skin and (D) lung homogenates of subcutaneous bleomycin-challenged mice. Each data point represents one animal, with bars representing the mean. For differences in Bleomycin challenged in comparison to naïve control groups * indicates p<0.05, **p<0.01, ***p<0.005 statistical significance.

Another model of fibrosis involves repeated subcutaneous delivery of bleomycin [24], which results in an experiment model of systemic sclerosis characterized by increased local skin collagen deposition, as well as a subsequent increase in pulmonary collagen deposition. This mode of bleomycin delivery results in a substantial increase in skin and lung collagen production. However, systemic rhBMP-7 treatment had no effect on the deposition of collagen at either site (Fig. 3A and B). Subcutaneous bleomycin administration also increased levels of the pro-fibrotic chemokine JE/CCL2 in both the skin and lung, and this too was not inhibited by rhBMP-7 treatment (Fig. 3C and D).

### BMP-7 has no effect on expression of profibrotic gene in human lung fibroblasts

**Figure 4:**
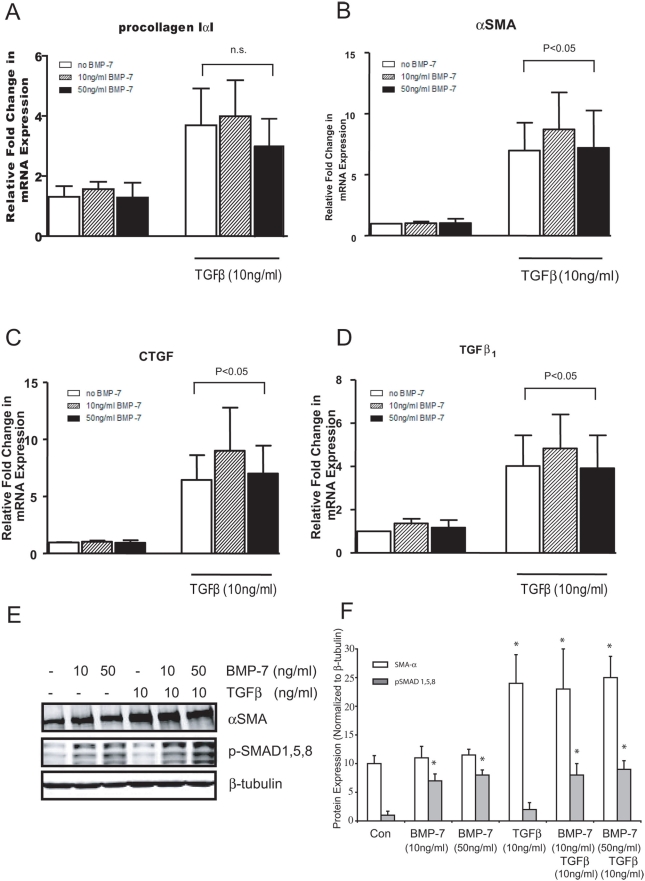
BMP-7 does not inhibit TGFβ_1_ induced gene expression in fibroblasts. Primary human lung fibroblasts (n = 3) were serum starved for 24 hours before incubation with rhBMP-7 at 10 or 50 ng/ml for one hour and then stimulated with rhTGFβ_1_ for 24 hours. Relative changes in mRNA expression of (A) procollagen-1, (B) αSMA, (C) CTGF and (D) TGFβ_1_ were analyzed using real-time RT-PCR. Protein expression of αSMA and phosphoSMAD 1,5,8, was (E) visualized and (F) quantitated to β-tubulin by Western blot analysis. * indicates p<0.05 significant difference in comparison to unstimulated cells.

Given that BMP-7 had little effect on indices of fibrosis *in vivo*, we asked whether BMP-7 affected fibroblast function *in vitro*. Exposure of human lung fibroblasts to TGFβ1 (10 ng/ml) upregulated expression of procollagen Iα1 (Fig. 4A), αSMA (Fig. 4B), CTGF (Fig. 4C) and TGFβ1 (Fig. 4D). Treatment with rhBMP-7 at either 10 or 50 ng/ml had no effect on expression of any of these genes, either alone or following treatment with TGFβ1 responses. We further evaluated αSMA at the protein level and found that BMP-7 did not modulate baseline or TGFβ-induced αSMA expression (Fig. 4E and F). Again phosphorylation of Smad 1,5,8 demonstrated that the exogenous rhBMP-7 was biologically active (Fig. 4E and F).

### BMP-7 does not affect TGFβ-induced epithelial to mesenchymal transition in lung epithelial cells

**Figure 5:**
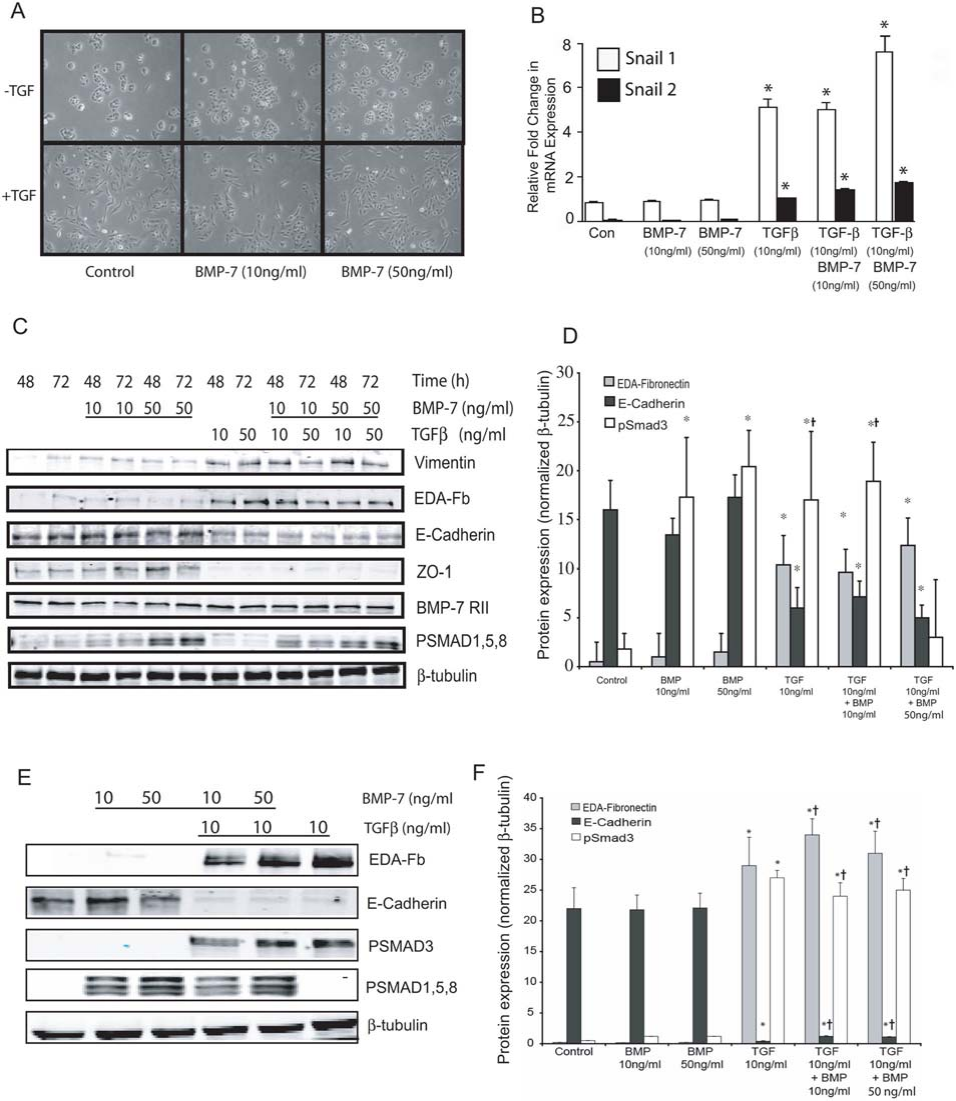
BMP-7 does not inhibit TGFβ_1_ induced EMT in A549 cells. A549 cells (n = 5) were serum starved for 24 hours before treatment with rhBMP-7 (10 or 50 ng/ml) for 24 hours followed by the presence or absence of rhTGFβ_1_ (10 ng/ml). (A) Phenotypic changes by light microscopy were noted in A549 cells cultured in medium with 0.5% serum and TGFβ_1_ (10 ng/ml) which were not attenuated by pre-incubation with BMP-7 at 10 or 50 ng/ml. (B) Snail-1 and Snail-2 gene expression was determined using real time RT-PCR in RNA isolated A549 cells after 48 hours of stimulation with BMP-7 (10 or 50 ng/ml) or TGFβ_1_ (10 ng/ml) alone or in combination, experiments were run in triplicate. (C) Western blot analysis of A549 lysates for the mesenchymal markers Fibronectin-EDA and vimentin, an epithelial markers E-Cadherin and ZO-1, BMP receptor II and phospho- Smad 1, 5, 8 expression following stimulation with BMP-7 alone or in combination with TGFβ_1_ for 48 or 72 hours. β-actin was used as a protein loading control. (D) Densitometric analysis of EDA-Fn ▒, E-Cadherin ▪ and phospho- Smad 1,5,8, □ expression shown in Figure 5C. (E) Western blot analysis of normal human bronchial epithelial lysates for Fibronectin-EDA, E-cadherin, phospho Smad 3, phospho Smad 1,5,8 expression following stimulation with BMP-7 alone or in combination with TGFβ_1_ for 72 hours. β-actin was used as a protein loading control. (F) Densitometric analysis of EDA-Fn ▒, E-Cadherin ▪ and phospho- Smad 1, 5, 8 □expression shown in Figure 5E.

Given that there appears to be some degree of differences in organ or cell specificity to BMP-7, we determined whether rhBMP-7 could influence TGFβ1-induced EMT in the human lung epithelial cell line A549. Incubation with TGFβ1 at either 10 or 50 ng/ml induced phenotypic changes consistent with EMT (Fig. 5A). Morphologically the cells lost their rounded cobblestone appearance and became stellate and migratory. Exposure to TGFβ1 also induced biochemical changes such as robust expression of the mesenchymal markers EDA fibronectin (EDA-FN) and vimentin, and a concomitant downregulation of the epithelial markers E-Cadherin and zonula occludens-1 (ZO-1) (Fig. 5B and C). Addition of rhBMP-7 alone had no obvious effect on the phenotype of A549 cells (Fig. 5A). When added at the same time as TGFβ1, rhBMP-7 (10 or 50 ng/ml) had no effect on cell morphology or expression of EDA-FN, vimentin, ZO-1 or E-Cadherin (Fig. 5B and C). BMP-7 signaling is mediated by BMP receptor II and subsequent recruitment and phosphorylation of Smad 1, 5 and 8. To confirm that BMP-7 was active, we evaluated BMP receptor II expression and Smad 1,5,8 phosphorylation by Western blot analysis. Comparable levels of BMPRII expression were seen under all conditions (Fig. 5B and C). Furthermore, BMP-7 induced a dose-dependent increase in phosphorylation of Smad 1,5,8 in the presence or absence of TGFβ1 (Fig. 5B and C) confirming that BMP-7 signaling is activated following TGFβ1 exposure. We also assessed whether BMP-7 interacted with TGFβ1 signaling, by examining phosphorylation of Smad 3 and expression of Smad 3-dependent genes Snail-1 and Snail-2 (slug). Exposure to rhBMP-7 had no effect on TGFβ1-induced expression of Snail-1 and -2 (Fig. 5D) or Smad 3 phosphorylation even when used at a supramaximal concentration of 500 ng/ml (data not shown). We extended these findings to evaluate the effect of rhBMP-7 on primary cultures of human bronchial epithelial cells and observed a similar phenomenon. In this setting, TGFβ1 induced EMT as described above. In contrast, BMP-7 had no effect on indices of EMT and did not modify the EMT responses to TGFβ1. This was not due to a lack of activity since we observed increased expression of phosphorylated Smad 1,5,8 in response to rhBMP-7 (Fig. 5E and F).

### Gene silencing of BMP-7 does not modulate TGFβ-induced EMT

**Figure 6:**
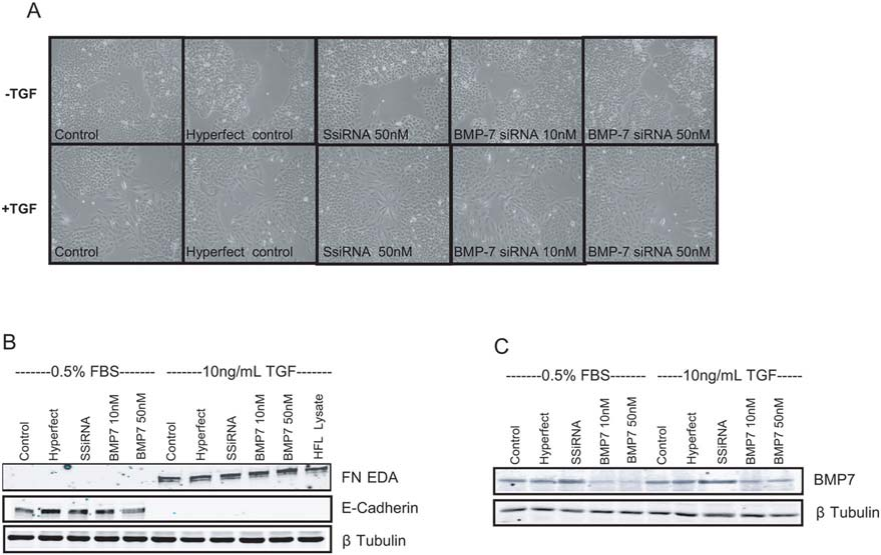
BMP-7 knock-down does not augment the ability of TGFβ_1_ to induced EMT in A549 cells. (A) Representative photomicrographs of A549 cultures (n = 3) transfected with BMP-7 siRNA (10 or 50 ng/ml), or non-silencing scrambled siRNA for 24 hours followed by the presence or absence of rhTGFβ_1_ (10 ng/ml) for 72 hours. (B) Western blot analysis of A549 cell lysates indicated increased expression of mesenchymal marker Fibronectin-EDA and loss of epithelial marker E-Cadherin following rhTGFβ_1_ treatment which was not enhanced following endogenous knock down of BMP-7. (C) Western blot analysis of BMP-7 showed efficient knock down of the protein following BMP-7 siRNA treatment.

To further confirm that BMP-7 does not influence TGFβ1-induced EMT, we used siRNA to knock down endogenous BMP-7 gene expression. As seen in Figure 6A and C, BMP-7 siRNA effectively decreased expression by >75%. However, despite this level of protein suppression, A549 cells still underwent EMT as judged by changes in morphology (data not shown) as well as decreased expression of E-Cadherin and increased expression of EDA-FN following exposure to TGFβ1 (Fig. 5B).

## Discussion

In the current study, we show that rhBMP-7: (1) did not affect bleomycin-induced fibrosis in either the lung or skin *in vivo*; (2) had no effect on expression of pro-fibrotic genes by human lung fibroblasts, either at rest or following exposure to TGFβ1; (3) did not modulate TGFβ1 -induced EMT in human lung epithelial cells *in vitro*. Taken together, these data suggest BMP-7 has little effect on fibrosis in the lung or the skin.

Previous studies have shown that bleomycin induces collagen deposition and that TGFβ1 plays an important role in mediating this process [3], [25]–[27]. In keeping with these findings, we found increased TGFβ1 expression that correlated with the increase in pulmonary collagen deposition following intra-tracheal bleomycin exposure. We also found increased BMP-7 protein expression following intratracheal bleomycin, however we hypothesized that the magnitude of BMP-7 induction is insufficient to modulate TGFβ1 activity in the lung. Therefore, to test this hypothesis we treated mice with daily injections of rhBMP-7 after bleomycin challenge. We chose a dose greater than previously demonstrated to be efficacious in murine models of fibrosis [22]. However, despite continual treatment for 14 days, the progression of lung fibrosis was not modified, suggesting that in lung cells, the inhibitory effects of BMP-7 are absent. Further, in our study there was increased IL-6 in the lungs of rhBMP-7 treated, bleomycin-challenged mice, indicating bioactive BMP-7. Also, immunohistochemical staining of lung sections from BMP-7 treated, bleomycin challenged animals showed increased phosphorylated Smad 1,5,8, indicating intact BMP-7 signaling. Therefore BMP-7, although active, was not therapeutic in our models of lung fibrosis at the efficacious doses used in this study.

In order to further test this hypothesis, we examined whether rhBMP-7 could ameliorate skin fibrosis induced by subcutaneous administration of bleomycin. However, as was seen in the lung fibrosis model, rhBMP-7 exerted no therapeutic benefit on collagen deposition in the skin. Given that subcutaneous bleomycin also results in lung fibrosis, akin to scleroderma-associated lung fibrosis [28]–[30], we examined the lungs of the mice involved in the dermal fibrosis study and found fibrosis that was indistinguishable from mice not exposed to rhBMP-7. Similarly, secretion of the pro-fibrotic chemokine JE/CCL2 in either lung or the skin was not modified by rhBMP-7. Based on this evidence we conclude that systemic BMP-7 treatment did not reduce lung or skin fibrosis.

Thus, while there is ample data to suggest that BMP-7 has potent anti-fibrotic effects on cells within the kidney [21], [31], the data from our current experiments suggests that BMP-7 has little effect in modifying fibrosis in either the lung or the skin. Indeed, the anti-fibrotic effects of BMP-7 in extra-renal tissues remain controversial. For example, Valcourt and colleagues undertook an extensive analysis of TGFβ1-induced EMT in several mouse and human epithelial cell lines [8]. The authors showed that except for αSMA expression, BMP-7 was ineffective at reversing TGFβ1 induced EMT. In our *in vivo* studies, we found that αSMA was the only gene evaluated whose expression trended towards an inhibition with rhBMP-7 treatment (data not shown) and this supported this finding. Tacke and co-workers [23] recently demonstrated that BMP-7 induced collagen and fibronectin production by hepatic stellate cells and was upregulated in the cirrhotic human liver. In contrast, Maric et al., showed that in rats with inflammatory bowel disease, systemic administration of BMP-7 led to less severe colitis with preserved histology and suppression of pro-inflammatory and pro-fibrogenic genes [32]. More recently, administration of rhBMP-7 was shown to inhibit the progression of cardiac fibrosis in mouse models of pressure overload hypertrophy and chronic allograft rejection [22]. The reasons underlying these dichotomous responses are unknown at present, but clearly warrant further investigation.

The anti-fibrotic effects of BMP-7 in renal, gastrointestinal and cardiac fibrosis have been associated with the ability of this protein to attenuate TGFβ1-induced epithelial or endothelial cell to mesenchymal cell transition [21], [22], [32]. We took advantage of these observations since EMT has been shown in lung epithelial cells from both mice and humans *in vitro* and also following bleomycin challenge *in vivo* [4], [13]. Exposure to TGFβ1 induced morphological characteristics of EMT, such as elongation and cell spreading and this was associated with phosphorylation of Smad 2/3. Addition of BMP-7 at 3 different concentrations was unable to modify these effects of TGFβ1. This was not due to lack of activity since we saw robust phosphorylation of Smad 1,5,8 in response to BMP-7. To investigate the mechanisms behind this lack of effect further we used two different strategies. Firstly we examined the effect of BMP-7 on TGFβ1-dependent Smad 2/3 activation and the Smad 3-dependent expression of Snail-1 and Snail-2. We show that BMP-7 does not influence Smad-3 phosphorylation nor the induction of Snail-1 and -2 alone or following TGFβ1 exposure. We confirmed this finding in primary cultures of normal human bronchial epithelial cells. In these cells, addition of rhBMP-7 had no effect on epithelial cell morphology or indices of cell signaling and did not modulate TGFβ1-induced EMT, despite inducing robust phosphorylation of Smad 1,5,8. In the final set of experiments, we used targeted siRNA to selectively knockdown BMP-7 expression. This strategy also had no effect on TGFβ1-driven EMT. These three independent streams of data provide further evidence for a lack of effect of BMP-7 in pro-fibrotic processes in lung epithelial cells. This hypothesis of tissue specificity is reinforced by the fact that we used a similar concentration range of BMP-7 (10 and 50 ng/ml) to that used by Zeisberg and colleagues [21], [33] to potently inhibit EMT in proximal tubular epithelial cells (10 and 100 ng/ml). Further evidence is provided by Kalluri and colleagues [22] in which effective doses of 1000 ng/ml of BMP-7 were required to inhibit endothelial-mesenchymal transition in the heart. However in our studies, supramaximal concentration of BMP-7 (500 ng/ml) also had no modulatory effect on TGFβ1-induced EMT.

Another cell intimately linked with the development of fibrosis is the fibroblast. In these cells, TGFβ1 mediates a variety of pro-fibrotic features including enhancing collagen production and growth factor synthesis [34], [35]. Consistent with the epithelial cell data, BMP-7 had no effect on these cells, either alone or following TGFβ1- mediated changes in gene expression.

Taken together we have shown that BMP-7 has no anti-fibrotic effect in lung fibrosis either at the *in vivo* or *in vitro* level. This suggests that the therapeutic options for BMP-7 may be confined to the renal compartment. This is supported by the fact that BMP-7 null mice die perinatally from renal failure [36].

## Materials and Methods

### Animal models of fibrosis

All experiments were conducted under Centocor IACUC regulations and protocols. Female C57Bl/6 mice (6–8 wk old) were purchased from ACE Laboratories (Boyertown, PA, USA). Mice were maintained in specific pathogen-free conditions and provided with food and water *ad libitum*. To induce intratracheal bleomycin-induced pulmonary fibrosis, mice were treated with intratracheal bleomycin (Blenoxane, Sigma, St. Louis, MO) on day 0, as previously described [37], [38]. Briefly, mice were anesthetized by intraperitoneal injection of 250 µl of 12.5 mg/ml ketamine followed by intratracheal instillation of 0.04 U of bleomycin in 50 µl of sterile PBS. Mice were analyzed for up to 21 days after bleomycin administration. Control animals received intratracheal sterile saline. For the studies evaluating the effect of BMP-7 on bleomycin-induced lung fibrosis, animals challenged with bleomycin were separated into two groups where they received vehicle control (20 mM acetate, pH 4.5 and 5% mannitol) or BMP-7 (500 µg/kg, i.p). For the subcutaneous bleomycin-induced skin and lung fibrosis model, mice backs were shaved and bleomycin administered subcutaneously daily for over 2 weeks. Control mice were injected daily with BMP-7 (500 µg/kg, i.p) or vehicle control (20 mM acetate, pH 4.5 and 5% mannitol) or PBS in the same anatomical site as the bleomycin mice. BMP-7 is well conserved between mouse and man (98% at the amino acid level [39]). Further, the recombinant human form has been used in several previous studies using murine models. Therefore in the present study, as in prior *in vivo* studies, we utilized human BMP-7 in our mouse models.

### Histology

Lungs were perfused *in situ* through the right ventricle with saline and then inflated under a constant pressure of 30 cm H_2_O with 1a ml of 10% normal buffered formalin (NBF). Lungs were ligated at the trachea, removed *en bloc*, and immersed in NBF for 24 hr. After which, tissue samples were changed to 70% alcohol before paraffin embedding, followed by sectioning, hematoxylin and eosin or Sirius Red staining. Sections were examined using a Fisher Scientific Micromaster inverted microscope and camera (Pittsburgh, PA, USA). For immunohistochemical analysis, five slides from each group containing 3 mice were stained separately to identify BMP-7 and phospho- Smad 1, 5, 8. Sections were de-paraffinized and rehydrated, and subject to antigen retrieval Subsequently, endogenous peroxidase was quenched with 3% H_2_O_2_ and blocked for 20 min with 50% goat serum. Primary mouse anti human BMP-7 (rabbit polyclonal, abcam, Cambridge, MA) and phospho- Smad 1,5,8 (rabbit polyclonal, Cell SignalingTechnology, Denvers, MA) or isotype control (normal rabbit IgG, Santa Cruz Biotechnologies, Santa Cruz, CA) were added overnight at 4°C in 25% goat serum. Sections were then incubated with a biotinylated goat anti-rabbit secondary antibody (Vector Labs Burlingame, CA) for 60 min followed by a 10 min treatment with Streptavidin-HRP (Dako, Mississauga, ON). The antigen of interest was visualized by using the brown chromogen 3,3-diaminobenzidine (Dako, Mississauga, ON) and counterstained with Harris Hematoxylin Solution (Sigma, Oakville, ON). Sections were then dehydrated and mounted with Cytoseal 60 (Richard-Allan Scientific, Kalamazoo, MI). Antibody dilutions and all washes were in TRIS-buffered saline solution.

For analysis all images were viewed and captured at 200× magnification. Five randomly selected digital 425×425 micron fields per slide were obtained with the use of a light microscope (Nikon Microphoto) equipped with a digital camera (JVC3-CCD KY F-70, Diagnostic Instruments). The same field was used to identify total cells and cells expressing BMP-7 and phosphor- Smad 1,5,8. Image analysis was performed using Image-Pro Plus (Media Cybernetics, Silver Spring, Maryland).

### Tissue preparation for protein and RNA analyses

For protein analysis, lungs from all groups of mice were homogenized in complete protease inhibitor (Roche Diagnostics Corp, Indianapolis, Indiana, USA) as previously described [37]. Homogenates were centrifuged at 900×g for 10 minutes and frozen at −80°C until time of analysis. For RNA analysis, lungs were removed, homogenized in PBS and RNA was isolated using RNeasy Plus Mini-Kits (QIAGEN, Valencia, CA). Purified RNA was treated with DNAse and reverse transcribed into cDNA using TaqMan® Reverse Transcription Reagents (Applied Biosystems, Foster City, CA). Gene expression was determined by real-time PCR using the Taqman® Universal PCR Master Mix (Applied Biosystems) and pre-developed Taqman® Gene Expression Assays designed by Applied Biosystems, *as per* manufacturer's instructions. Fold induction was determined after calibration of the gene of interest with 18S rRNA and normalization to either saline control gene expression or for the effect of BMP-7 treatment, after normalization to vehicle control bleomycin-challenged mice.

For skin fibrosis studies, on the day after the final bleomycin injection, the skin at the injection site was removed by using an 8 mm dermal biopsy punch (Miltex, York PA) and processed for protein or RNA as described above.

### Sircol Collagen assay

The Sircol collagen assay (Biocolor Ltd., Belfast, Ireland) was performed following the manufacturer's instructions [40]. Briefly, Sirius red reagent was added to each lung homogenate (50 µl) and mixed for 30 minutes at room temperature. The collagen–dye complex was precipitated by centrifugation at 16,000 *g* for 5 minutes and the pellet was resuspended in 1 ml of 0.5 M NaOH. The concentration of collagen in each sample was measured as absorbance at 540 nm and values extrapolated from a known standard curve *as per* manufacturer's instructions. Chemokine levels were measured using luminex technology (Biosource, Invitrogen, Carlsbad, CA).

### Fibroblast and epithelial protein and gene expression

Pulmonary fibroblasts were isolated from lung tissue obtained from human transplant donor lungs deemed unsuitable for transplantation (n = 4) and donated for medical research obtained through the International Institute for the Advancement of Medicine (Edison, NJ). The ethic committees of the involved institutions approved this study. All fibroblast cell lines were isolated by serial passaging and purity assessed using morphological and immunohistochemical staining as previously described [41]. Fibroblasts were plated into 6 well plates (Costar, Corning, NY) at 1×10^5^ cells/well and allowed to adhere for 8 hours. The cells were then washed with PBS and cultured overnight in serum free DMEM media containing 1% penicillin and 1% streptomycin at 37°C in 5% C0_2_/air. Cells were then stimulated with or without recombinant human BMP-7 (rhBMP-7; RnDSystems, Minneapolis, USA) or TGFβ_1_ (Santa Cruz Biotechnology, USA), or both combined at 10 and 50 nM concentrations for 24 hrs. Supernatants were removed and RNA was subsequently isolated and analyzed by real-time PCR as described above. Here, fold induction was determined after calibration of the gene of interest with 18S rRNA and normalized to corresponding unstimulated cells. Epithelial Snail 1 and Snail 2 gene expression was evaluated in A549 cells stimulated with BMP-7 or TGFβ1 alone or in combination. Protein levels of αSMA and phospho- Smad 1,5,8 were analyzed by Western analysis and normalized to β-tubulin which served as the loading control.

### EMT Analysis

Experiments were performed in the A549 cell line and normal bronchial epithelial cells (NHBE; Lonza Canada Inc, Shawinigan) both grown to 60% confluence in 6 well plates (Costar, Corning, NY). A549 cells were grown in DMEM containing 10% fetal bovine serum and NHBE cells grown in bronchial epithelial growth media (BEGM, Cambrex, Lonza Canada Inc). All cultures were maintained at 37°C, 5% CO_2_/air. Prior to each experiment, cells were incubated in respective serum free media for 24 hrs. A549 and NHBE cells were then incubated with or without rhBMP-7 or TGFβ_1_ or both combined at 10 and 50 nM concentrations for 48 and 72 hrs. Following this, cells were lysed in complete protease inhibitor (Roche Diagnostics Corp). Lysates at a concentration of 50 ng/ml were then electrophoresed in SDS-polyacrylamide gels, electrotransferred to nitrocellulose, and membranes underwent Western analysis with antibodies directed against fibronectin-EDA (MAB1940,Chemicon International, Temecula, USA), E-Cadherin (Clone G 10, Santa Cruz Biotechnology, USA); phospho- Smad 1,5, & 8, (AB3848) BMP receptor II (MAB3551), and rhBMP-7 (RnD Systems). β-tubulin (Upstate, Lake Placid, NY) was used and protein loading control.

### BMP-7 knock-down with SiRNA

A549 cells grown to 50% confluence in six well plates were transfected with ON-TARGET plus SMART pool BMP-7 SiRNA (Dharmacon, Chicago, USA) or non-silencing control siRNA at a final concentration of 10 or 50 nM using Hyperfect (Quiagen, USA) in DMEM containing 10% fetal bovine serum at 37°C, 5% C0_2_. After 24 hrs, cells were incubated in DMEM containing 0.5% serum for a further 24 hrs, and then incubated with TGFβ_1_at 10 nM for 48 hrs. Cells were then lysed and analyzed by Western blot as above.

### Statistical analysis

All statistical analysis were calculated using GraphPad Prism software (San Diego, CA). All statistical comparisons were performed using nonparametric Mann Whitney t-test. Data was considered significant if p<0.05, p<0.01 or p<0.005, which is referred to as *, ** or *** respectively.
